# Quantitative interocular comparison of total corneal surface area and corneal diameter in patients with highly asymmetric keratoconus

**DOI:** 10.1038/s41598-022-08021-6

**Published:** 2022-03-11

**Authors:** François-Xavier Crahay, Guillaume Debellemanière, Stephan Tobalem, Wassim Ghazal, Sarah Moran, Damien Gatinel

**Affiliations:** 1 Rothschild Foundation Hospital, Paris, France; 2 CHR Citadelle, Liège, Belgium

**Keywords:** Corneal diseases, Eye manifestations, Medical research

## Abstract

Keratoconus is a progressive corneal disorder which is frequently asymmetric. The aetiology of keratoconus remains unclear, and the concept of keratoconus as an ectatic disorder has been challenged recently. We carried out a retrospective study in 160 eyes of 80 patients, to evaluate and compare interocular differences in corneal diameter and surface area in patients with unilateral or highly asymmetric keratoconus (UHAKC). Calculations were performed using raw topographic elevation data derived from topographic measurements using Orbscan II, and we extrapolated surface areas up to measured corneal diameter. We also evaluated inter-eye correlation, and correlation between corneal surface area, corneal diameter and keratoconus severity. Our results showed a statistically significant but not clinically important greater corneal diameter (12.14 mm and 12.17 mm; p = 0.04), and corneal surface area (paired t-test, p < 0.0001; p = 0.0009 respectively) in more affected eyes. Inter-eye comparison revealed corneal diameter, anterior chamber depth, and corneal surface area were strongly correlated between eyes. Corneal surface area remained strongly correlated, and Bland–Altman analysis also showed strong inter-ocular agreement. Our results show that in patients with UHAKC the interocular difference in corneal diameter and corneal surface area is clinically insignificant, and are consistent with a redistribution, rather than increase, of corneal surface area with keratoconus progression.

## Introduction

Keratoconus is an ocular condition defined by progressive corneal thinning, steepening and irregular astigmatism, often leading to visual impairment^1,2^.

Keratoconus is a highly prevalent disease; however the pathophysiology is not yet fully understood. In recent papers, we proposed a primarily mechanical cause^3–9^, with eye rubbing playing a fundamental role, with other known risk factors (biochemical, genetic, environmental) likely being associated with eye rubbing.

Recent studies that we conducted in keratoconus revealed that anterior and posterior corneal surface areas were slightly greater in eyes with keratoconus, but that surface areas did not increase with keratoconus severity^10^. However, we also found greater horizontal corneal diameter in keratoconus eyes, thus potentially inducing a bias in the corneal surface area calculation. In this paper, we report an interocular comparison in patients with unilateral or highly asymmetric keratoconus. We recently published a case–control study of consecutive Unilateral or Highly Asymmetric Keratoconus (UHAKC) patients diagnosed at the Rothschild Foundation which revealed an association between eye rubbing, incorrect sleeping position and UHAKC^7^, with eye rubbing and incorrect sleeping position found more frequently in the worse eye.

Corneal surface area was calculated using raw topographic elevation data in 80 patients with UHAKC.

As corneal diameter has been shown to be symmetric in normal myopic eyes^11^, we studied symmetry of corneal diameter and corneal surface areas in highly asymmetric keratoconus.

We also evaluated inter-eye correlation, and correlation between corneal surface area, corneal diameter, and keratoconus severity.

## Results

### Demographic data

Our study involved 160 eyes in 80 patients with UHAKC. We enrolled 15 women and 65 men.

Table 1 summarises demographic characteristics.

**Table 1:** Demographics characteristics of enrolled patients (*SD* standard deviation).

Demographic characteristics	Highly asymmetric keratoconus
Patients (n)	80
**Age (years)**	
Mean ± SD	28.63 ± 11
Range	13–70
**Gender**	
Female (n, %)	15 (18.8%)
Male (n, %)	65 (80.3%)

All patients had unilateral or highly asymmetric keratoconus (UHAKC) as defined in a prior study^7^. The right eye was either unaffected, or less affected in 35 patients (43.8%), as summarized in Table 2.

**Table 2:** Proportion of affected eye per side.

Side	Less or unaffected eye	Affected eye
OD (n, %)	35 (43.8%)	45 (56.3%)
OS (n, %)	45 (56.3%)	35 (43.8%)

As expected, affected eyes had thinner (446.3 µm and 511.7 µm; p < 0.0001; R squared (R^2^) = 0.80) and steeper corneas (50.80 diopters and 43.44 diopters; p < 0.0001; R^2^ = 0.70). Internal anterior chamber was deeper (3.39 mm and 3.19 mm; p < 0.0001; R^2^ = 0.76) in affected eyes.

Corneal diameter was statistically slightly greater in affected eyes (12.14 mm and 12.17 mm; p = 0.04, R^2^ = 0.05, Table 3).

**Table 3:** Mean topographic parameters for less or unaffected eyes and affected eyes.

Topographic parameters: mean ± SD	Less or unaffected eye	Affected eye	*p*-value*
Minimal pachymetry (µm)	511.7 ± 28	446.3 ± 46	< 0.0001
Maximal keratometry (D)	43.44 ± 0.92	50.80 ± 4.84	< 0.0001
Internal anterior chamber depth from endothelium(mm)	3.19 ± 0.32	3.38 ± 0.31	< 0.0001
Corneal diameter (mm)	12.14 ± 0.33	12.17 ± 0.35	0.04
Total anterior surface area (mm^2^)	136.7 ± 8.15	138.3 ± 8.79	< 0.0001
Total posterior surface area (mm^2^)	146.2 ± 9.20	147.8 ± 10.13	0.0009

Anterior (138.3 cm^2^ and 136.7 cm^2^; p < 0.0001, R^2^ = 0.17) and posterior (147.8 cm^2^ and 146.2 cm^2^; p = 0.0009, R^2^ = 0.13) corneal surface areas were significantly greater in affected eyes.

Analysis of correlation.

Pearson r correlation coefficient is shown in Table 4 (affected eyes) and Table 5 (less or unaffected eyes). As expected from a geometrical standpoint, corneal diameter was strongly correlated with anterior and posterior corneal surface areas, in both the affected and less or unaffected eye groups. Anterior chamber depth was weakly correlated with corneal diameter and corneal surface areas.

**Table 4:**
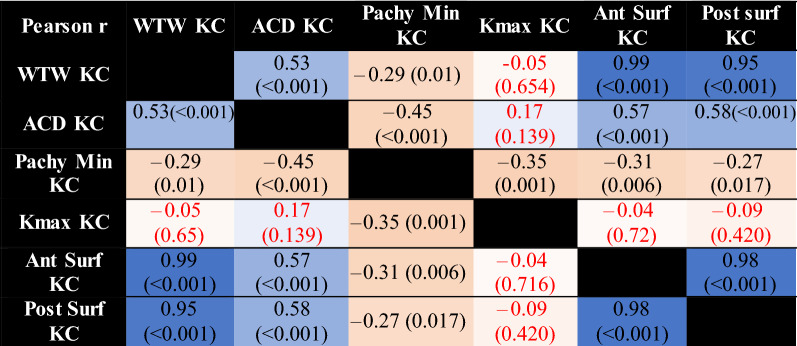
Pearson r correlation coefficient correlation matrix in affected eyes.

The darker the colour of the highlighted cell, the stronger the correlation. Non statistically significant results (p > 0.05) are in red. (*KC* affected eye, *N* less or unaffected eye, *WTW* horizontal corneal diameter, *ACD* internal anterior chamber depth, *Pachy min* minimal pachymetry, *Kmax* maximal keratometry, *Ant Surf* anterior surface area, *Post Surf* posterior surface area).

**Table 5:**
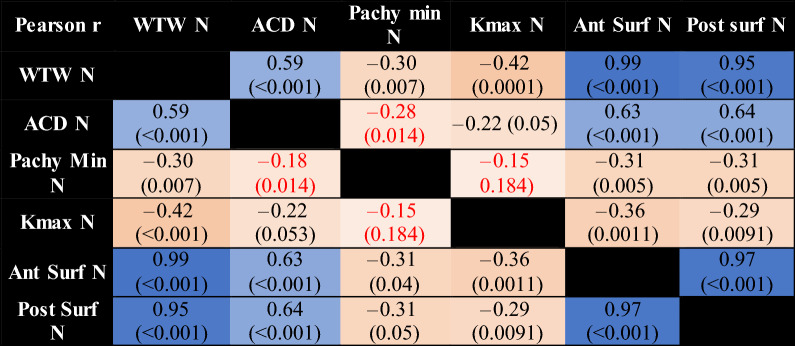
Pearson r correlation coefficient correlation matrix in less affected or unaffected eyes.

The darker the colour of the highlighted cell, the stronger the correlation. Non statistically significant results (p > 0.05) are in red. (*KC* affected eye, *N* less or unaffected eye, *WTW* horizontal corneal diameter, *ACD* internal anterior chamber depth, *Pachy min* minimal pachymetry, *Kmax* maximal keratometry, *Ant Surf* anterior surface area, *Post Surf* posterior surface area).

Pearson r correlation coefficient between affected and less or unaffected eyes is reported in Table 6. Minimal pachymetry and maximal keratometry had a weak inverse correlation with corneal surface areas. Inter-eye comparison showed that corneal diameter, anterior chamber depth, corneal surface areas were strongly correlated between eyes. Minimal pachymetry showed slight correlation between eyes. On the other hand, maximal keratometry did not demonstrate any correlation between eyes, as expected in UHAKC.

**Table 6:**
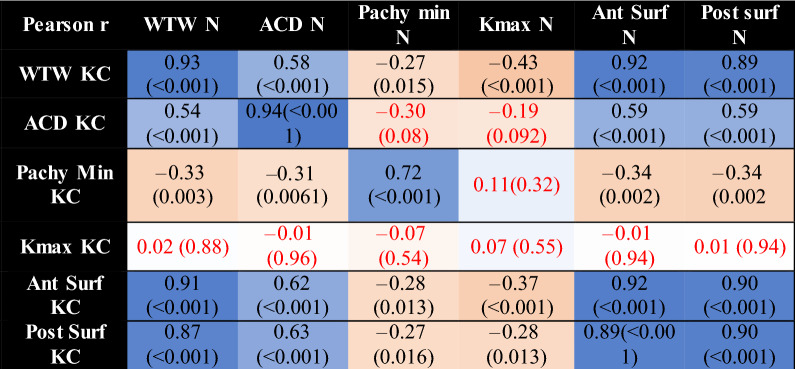
Pearson r correlation coefficient correlation matrix between less affected or unaffected eyes and affected eye.

The darker the colour of the highlighted cell, the stronger the correlation. Non statistically significant results (p > 0.05) are in red. (*KC* affected eye, *N* less or unaffected eye, *WTW* horizontal corneal diameter, *ACD* internal anterior chamber depth, *Pachy min* minimal pachymetry, *Kmax* maximal keratometry, *Ant Surf* anterior surface area, *Post Surf* posterior surface area).

Bland–Altman analysis

**Figure 1:**
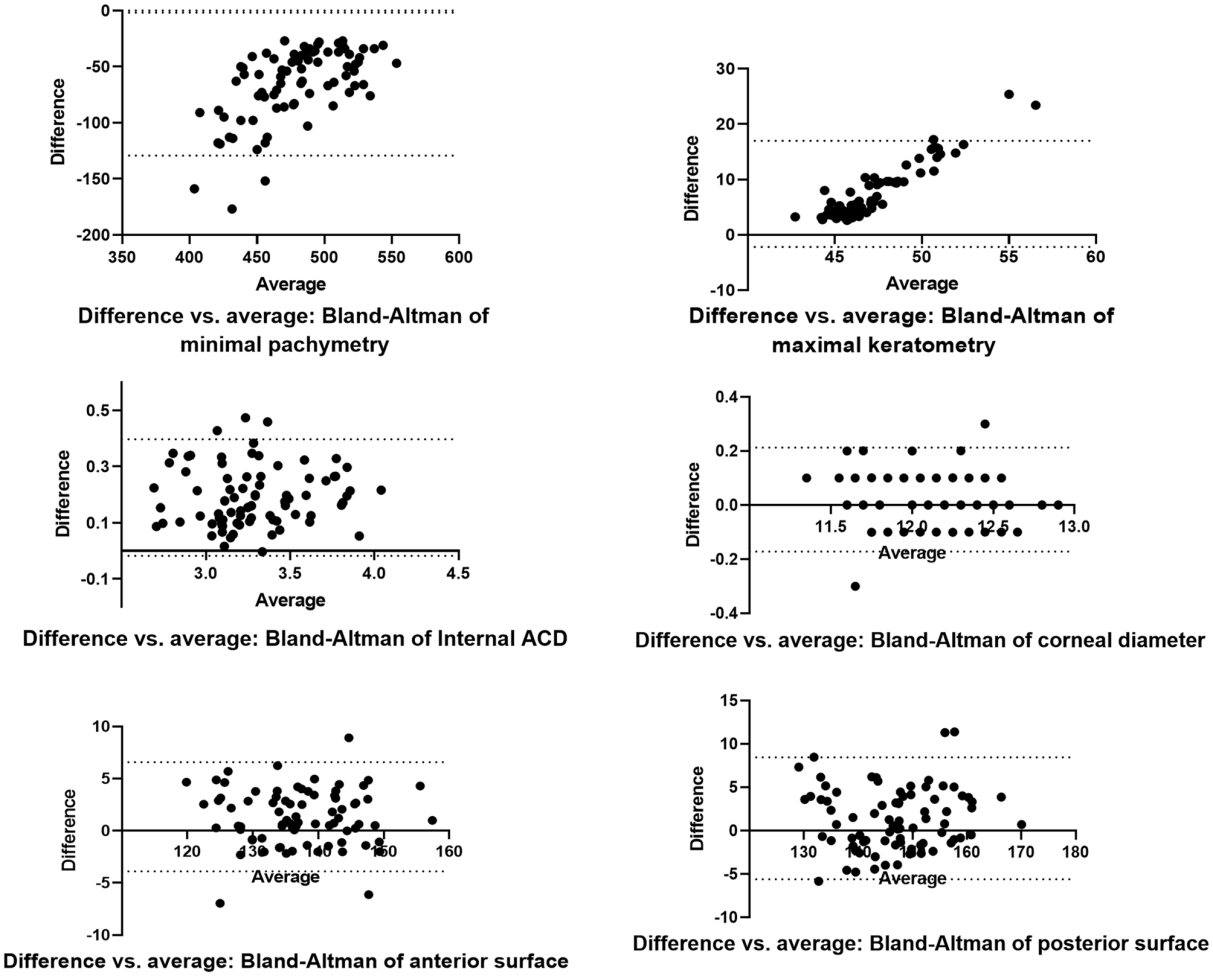
Bland–Altman graphs plotting difference between unaffected eyes and affected eyes and average of unaffected eyes and affected eyes. Dotted line represents 95% of agreement.

The Bland–Altman graphs (Fig. 1) show the agreement between the two eyes for the different parameters. As expected in patients with UHAKC, the maximum keratometry (95% limits of agreement − 2.2 to 16.97) and minimum pachymetry (95% limits of agreement − 129.3 to 1.70) were highly asymmetric. The internal anterior chamber depth also showed a statistically significant asymmetry (95% limits of agreement − 0.02 to 0.40). In contrast, no significant inter-ocular difference is seen in either corneal diameter (95% limits of agreement − 0.22 to 0.29) or corneal surface area (anterior: 95% limits of agreement − 5.26 to 8.46; posterior: 95% limits of agreement − 6.87 to 10.25).

## Discussion

The concept of keratoconus as a true corneal ectasia is being increasingly challenged in recent studies. The term ectasia, implies stromal stretching; an anisometric process which should result in an increase of the surface area. However, studies on this subject are conflicting. Using raw data from Placido specular corneal topography, Smolek and Klyce^12^ analysed anterior corneal surface area in normal corneas and keratoconus. They concluded that corneal surface area was similar in both groups, which strongly suggested keratoconus as a form of warpage rather than a true ectasia. They also followed a case showing no area increase at all with keratoconus progression. More recently, Kitazawa et al., as well as Cavas-Martinez et al., found greater central corneal surface area in keratoconus^13,14^ using data from anterior and posterior corneal surface areas obtained with an anterior segment OCT and a Sirius system corneal topographer respectively. However, it is important to note that those studies used particular geometrical assumptions and algorithms, rather than direct elevation based discrete surface element summing^15^ for calculating the corneal surface area, and did not take the total diameter of the cornea into account.

In the present study, we challenged our previous findings excluding potential inter-individual biases. To calculate corneal surface area, we used a previously described method inspired from landscape measurements, using raw elevation data with no assumption on the corneal shape or local curvature^15^. We obtained elevation data from topographic measurements of Orbscan II which has been shown to be repeatable^16^ and we extrapolated surface areas up to measured corneal diameter.

We found that, although anterior and posterior corneal surface area was slightly greater in affected eyes, it is unlikely that this difference reflects a true ectatic process (difference between the means of 1.6 mm^2^ which represents an increase of about 1.17% and 1.09% of the anterior and posterior corneal surfaces, respectively). The effect size is also moderate for surface area. Moreover, corneal surface area remained strongly correlated, and Bland–Altman analysis also showed a strong inter-ocular agreement. Our results show, that in the same individual suffering from a UHAKC, there was a statistically significant, although clinically irrelevant difference, between the affected and less or unaffected eye. If it was assumed that both corneas exhibited a high degree of enantiomorphism before the onset of unilateral keratoconus, then the small difference measured after the onset of keratoconus reflects a different process from true ectasia. Epithelial remodelling could probably influence the anterior area of the cornea^17^ and contribute to corneal surface modifications.

In addition, surface area showed greater correlation with corneal diameter and anterior chamber depth than with maximal keratometry and minimal pachymetry, and the effect size is medium regarding corneal surface area. In our opinion, these results demonstrate that it is unlikely that increased corneal surface area is a sign of keratoconus progression or keratoconus severity, and is not consistent with increasing surface area from a stromal stretching effect in progressive keratoconus. Further studies regarding corneal volume and anterior chamber volume could provide more information. Rather, the data suggests that a progressive surface area increase resulting from stromal stretching as keratoconus progresses seems inconsistent. Corneal surface area and corneal diameter differences observed in patients with highly asymmetric keratoconus are consistent with our previous results between normal eyes and keratoconus. These observations suggest that keratoconus corresponds to a corneal surface area redistribution rather than any significant corneal stretching. It more likely represents a permanent corneal deformation with a relatively stable total surface area, and therefore may be considered as an extreme form of corneal warpage caused by structural damage secondary to stromal degeneration and external forces^12^. We have previously shown that unilateral eye-rubbing and nocturnal compression on the same side can lead to UHAKC^7^. In this case–control study, we found that patients with unilateral or highly asymmetric keratoconus reported significantly more eye rubbing and more rubbing in the affected eye. Sleeping on the side or stomach was also found to be a risk factor for developing keratoconus. Surprisingly, we did not find an increased risk in the presence of a family history of keratoconus.

We measured a slight increase in the depth of the anterior chamber on the affected side. In the context of a generally isometric deformation, the average curvature of the corneal surfaces is largely unaffected, which implies that an increase in the paracentral curvature is necessarily accompanied by a decrease in the peripheral curvature, and an increase in the negative asphericity of the corneal profile. A central sagittal curvature steepening with a concomitant peripheral flattening could explain a slight central deepening of anterior chamber. The entire anterior chamber volume could be studied to clarify this finding.

In light of our previous study^10^ which showed greater corneal diameter in keratoconus than normal eyes, we aimed to establish the role of horizontal corneal diameter in the surface area differences we observed. We found in UHAKC that corneal diameter was slightly greater in the affected eye, however this difference was small and at the limit of statistical significance. Moreover, Bland–Altman analysis showed strong agreement between the keratoconus eye and the fellow eye. We thus hypothesised that it seems unlikely that corneal diameter is a marker or a consequence of severity and progression of keratoconus. To explain the presence of a larger corneal diameter in patients with keratoconus, we can hypothesize that a larger corneal diameter offers less mechanical resistance due to reduced scleral support. More studies are needed to clarify the possible role of corneal diameter in keratoconus pathogenesis.

## Methods

### Patients

This retrospective study included subjects examined at the Department of Anterior Segment and Refractive surgery at Rothschild Foundation Hospital, Paris, France. The study and data collection were achieved with approval from the Rothschild Foundation Institutional Review Board which followed the tenets of the Declaration of Helsinki. Informed consent was obtained from all subjects prior to collection of their data.

Inclusion criteria for unilateral or highly asymmetric keratoconus were patients with keratoconus in one eye and normal corneal topography in the fellow eye. As previously reported in our study^7^, we used a combination of quantitative videokeratography-derived indices. We used Score (Score Analyser, Orbscan)^18,19^ and Belin-Ambrosio Display Diagnosis index (BAD-D) (Topolyzer)^20^ to identify Unilateral or highly asymmetric keratoconus (UHAKC)^21^. Normal topography was defined as Score < 1.5 and BAD-D < 1.4 and Keratoconus was defined ad Score > 4 and BAD-D > 5.0. UHAKC was defined as patients with one normal eye and one keratoconus eye.

Exclusion criteria in both groups were any previous ocular surgery (e.g. penetrating keratoplasty, lamellar keratoplasty, corneal rings, corneal collagen cross-linking) and any other ocular disease. We excluded patients with superficial corneal lesions such as superficial punctate keratitis and epithelial erosions. Patients using rigid gas permeable contact lenses were also excluded. Patients using soft contact lenses were asked to stop wearing them two weeks before the examination. Topographies with missing data within the central 8 mm were excluded.

First, elevation maps obtained by Orbscan II at Rothschild Foundation Hospital were classified using a machine learning algorithm^22^ as keratoconus or normal. We obtained 7003 elevation maps from 2041 keratoconus without any missing data within the central 8 mm. We kept a single elevation map for each eye. Topographies were manually checked for inclusion and exclusion criteria.

We finally only selected bilateral asymmetric keratoconus leaving 160 eyes from 80 patients.

### Corneal topography method

Topography was obtained using the Orbscan II corneal topographer (Bausch & Lomb). This device uses a Placido disk with 40 rings combined with 40 scans of slit lamp acquisition. Scans were obtained by qualified technicians using standard protocol for Orbscan topography acquisition. Poor quality exams were repeated. The Orbscan’s software provided anterior and posterior elevation maps including 10,000 measurements for a corneal zone of 10 by 10 mm. We used the raw data, without taking into account the sphere of reference which is used for the representation of the elevation maps^23^.

Orbscan II also provided biometric and keratometry values: corneal diameter (horizontal white to white), minimal pachymetry, maximal keratometry, and cornea irregularities in the central 3.0 mm diameter and the 3 to 5 mm ring.

All topographies were acquired at Rothschild Foundation Hospital. Data was exported from Orbscan and analysed and classified using NumPy v1.18.0 package.

### Corneal surface calculation

Corneal surface area was calculated from anterior and posterior elevation raw data using a method previously described for landscape surface area calculation^15^. We have already detailed the technique in our previous study^10^.

### Data analysis

Data was compiled in Microsoft Excel files. Variance analysis and descriptive statistics were performed with Microsoft Excel and Prism GraphPad software. Normality tests were performed for each parameter using the Shapiro–Wilk normality test. Since the data were normally distributed, we used a paired t-test. We analyse correlation between variables using Pearson correlation test. P-value was determined for each correlation.

A P value of less than 0.05 was considered statistically significant. Effect size was estimated using R squared (R^2^) or partial eta squared.

Bland–Altman analysis and 95% limits of agreement were calculated using GraphPad software.
